# Eravacycline in Critically Ill Patients with Multidrug-Resistant Gram-Negative Infections: A Real-World Cohort Study

**DOI:** 10.3390/jcm15145631

**Published:** 2026-07-17

**Authors:** Stelian Adrian Ritiu, Adelina Baloi, Marius Papurica, Dorel Sandesc, Daiana Toma, Sonia Elena Popovici, Claudiu Rafael Barsac, Madalina Butaș, Ana-Maria Botoaca, Ovidiu Horea Bedreag

**Affiliations:** 1 Faculty of Medicine, “Victor Babes” University of Medicine and Pharmacy, 300041 Timisoara, Romaniapopovici.sonia@umft.ro (S.E.P.);; 2 Clinic of Anaesthesia and Intensive Care, Emergency County Hospital “Pius Brinzeu”, 300723 Timisoara, Romania; 3 Doctoral School, “Victor Babes” University of Medicine and Pharmacy Timisoara, Eftimie Murgu Square 2, 300041 Timisoara, Romania; 4 Anaesthesia and Intensive Care Research Center (CCATITM), “Victor Babes” University of Medicine and Pharmacy, 300041 Timisoara, Romania

**Keywords:** eravacycline, multidrug-resistant infections, critical care, salvage therapy, mortality, carbapenem-resistant infections, ICU, microbiological eradication

## Abstract

**Background:** Multidrug-resistant Gram-negative infections remain a major challenge in critically ill patients, particularly when caused by carbapenem-resistant pathogens with limited therapeutic options. Although eravacycline has emerged as a potential salvage agent, real-world data in high-acuity intensive care populations remain scarce. **Methods:** We conducted a retrospective cohort study including adult ICU patients who received eravacycline as salvage therapy for multidrug-resistant Gram-negative infections between February 2024 and September 2025 in a tertiary-care center. Demographic, clinical, microbiological, and treatment-related variables were extracted from electronic records. Infection complexity was classified as monomicrobial single-site, monomicrobial multi-site, or polymicrobial. The primary outcome was in-hospital mortality; secondary outcomes included clinical response, microbiological eradication, and safety. **Results:** Forty critically ill patients were included (median age 58 years, IQR 47–69; mean APACHE II 16.4 ± 6.9; mean SOFA 5.5 ± 3.3). Pulmonary (62.5%) and intra-abdominal (55.0%) infections were most frequent. *Klebsiella pneumoniae* predominated (75.0%), followed by *Acinetobacter baumannii* (35.0%) and *Pseudomonas aeruginosa* (22.5%). Most patients received combination antimicrobial therapy. Microbiological eradication or presumed eradication was documented in 24 patients (60.0%), yet overall in-hospital mortality remained high (75.0%). Baseline disease severity and persistent inflammatory response were more strongly associated with unfavorable outcomes than treatment timing or duration. **Conclusions:** In this real-world ICU cohort, eravacycline-based salvage therapy was associated with moderate microbiological response but very high mortality, reflecting the severity of underlying critical illness. The dissociation between microbiological eradication and survival suggests that outcomes are more closely related to host factors and unresolved systemic inflammation than to pathogen clearance alone. Eravacycline remains one of the few agents active against MDR Gram-negative pathogens (with no activity against *Pseudomonas aeruginosa*), frequently used off-label; larger controlled studies are needed to establish efficacy beyond complicated intra-abdominal infections.

## 1. Introduction

The escalating prevalence of multidrug-resistant (MDR) Gram-negative pathogens, particularly carbapenem-resistant *Enterobacterales* (CRE) and *Acinetobacter baumannii* (CRAB), represents a major challenge in intensive care settings. The widespread dissemination of resistance mechanisms such as carbapenemase production (e.g., KPC, OXA-48 and metallo-beta lactamases like NDM and VIM) has led to severely limited therapeutic options, while the selective pressure exerted by broad-spectrum antibiotics further drives the emergence of highly resistant strains. These infections are associated with high mortality rates, particularly in critically ill patients with significant comorbidities and organ dysfunction [1,2].

Eravacycline, a synthetic fluorocycline antibiotic, has demonstrated in vitro activity against a broad spectrum of MDR Gram-negative pathogens (excluding *Pseudomonas aeruginosa*) and has been approved for the treatment of complicated intra-abdominal infections. While clinical trials have shown favorable efficacy in non-critically ill populations, real-world data in ICU patients—particularly in the context of salvage therapy for advanced infections—remain limited. In such settings, eravacycline is often used after failure of multiple prior antimicrobial regimens, in patients with high disease severity and complex infection profiles [3,4].

Importantly, the relationship between microbiological response and clinical outcomes in critically ill patients with MDR infections remains poorly understood. While pathogen eradication is traditionally considered a key therapeutic goal, increasing evidence suggests that survival may be more strongly influenced by host factors, including baseline severity of illness and the ability to resolve systemic inflammation. The heterogeneity of ICU populations further complicates the interpretation of treatment response, as patients with similar microbiological profiles may experience markedly different clinical trajectories [5,6].

In this context, the present study aimed to characterize clinical outcomes, microbiological response, and determinants of mortality in a real-world cohort of critically ill patients treated with eravacycline as salvage therapy for MDR Gram-negative infections. Additionally, we sought to explore patient heterogeneity using data-driven approaches, including clustering and predictive modeling, to better understand the interplay between pathogen burden, host response, and clinical outcomes.

## 2. Materials and Methods

### 2.1. Study Design and Setting

This retrospective observational cohort study was conducted at “Pius Brînzeu” Emergency County Clinical Hospital Timișoara, Romania, a 1198-bed tertiary care academic medical center with a 44-bed intensive care unit specializing in mixed (medical and surgical) critical care. The study was conducted in accordance with the Declaration of Helsinki and approved by the Institutional Review Board of the “Pius Brînzeu” Emergency County Clinical Hospital Timișoara (approval number 569, 8 October 2025) and by the Institutional Ethics Committee for Scientific Research of the “Victor Babeș” University of Medicine and Pharmacy Timișoara (approval number 86/2025). The requirement for informed consent was waived due to the retrospective nature of the analysis.

### 2.2. Study Population

#### 2.2.1. Inclusion Criteria

We included all adult patients (age ≥ 18 years) admitted to the ICU between February 2024 and September 2025 who met the following criteria:

- Microbiologically confirmed infection with multidrug-resistant organisms;
- Receipt of eravacycline for at least 48 h;
- Complete medical records available for review;
- Availability of baseline severity scores (APACHE II and SOFA) at ICU admission.

#### 2.2.2. Exclusion Criteria

Patients were excluded if:

- Age < 18 years;
- Eravacycline treatment duration < 48 h (to exclude brief trial and immediate discontinuation);
- Incomplete medical records precluding assessment of primary outcomes;
- Transfer from another institution with >72 h of ICU care prior to admission (to ensure accurate baseline assessment).

**Figure 1:**
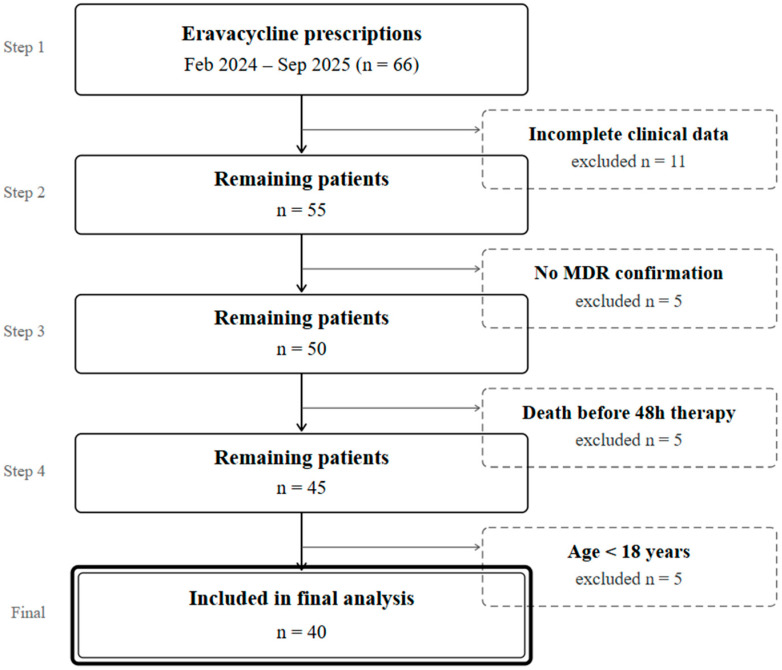
CONSORT-style flow diagram illustrating the stepwise selection of patients included in the final analysis. Of 66 critically ill patients who received eravacycline during the study period (February 2024–September 2025), 26 were excluded based on predefined criteria: incomplete clinical data (*n* = 11), absence of microbiological confirmation of multidrug-resistant infection (*n* = 5), death within 48 h of treatment initiation (*n* = 5), and age under 18 years (*n* = 5). A total of 40 patients met all inclusion criteria and were included in the final analysis. MDR, multidrug-resistant.

The stepwise patient selection process is illustrated in Figure 1. Of 66 patients who received eravacycline during the study period, 26 were excluded based on predefined criteria: incomplete clinical data (*n* = 11), absence of microbiological confirmation of MDR infection (*n* = 5), death within 48 h of treatment initiation (*n* = 5), and age under 18 years (*n* = 5), yielding a final cohort of 40 patients.

#### 2.2.3. Patient Identification

Eligible patients were identified through review of the hospital pharmacy database for all eravacycline prescriptions during the study period. Medical records were then manually reviewed to confirm eligibility and extract study variables.

### 2.3. Data Collection

#### 2.3.1. Baseline Characteristics

Demographic data collected included age, sex, weight, and comorbidities. Disease severity was assessed using the Acute Physiology and Chronic Health Evaluation II (APACHE II) score and Sequential Organ Failure Assessment (SOFA) score, calculated using worst values within the first 24 h of ICU admission. Clinical variables included presence of acute respiratory distress syndrome (ARDS, defined by Berlin criteria), vasopressor use, mechanical ventilation requirement and duration, and surgical interventions categorized as abdominal, brain, or other surgery. APACHE II and SOFA scores were calculated at ICU admission for all patients. Arterial blood gas analysis (ASTRUP) is performed as a mandatory institutional protocol for all ICU admissions, and APACHE II calculation is an obligatory component of the admission assessment. Consequently, no missing data were recorded for any physiological variable required for score computation.

#### 2.3.2. Infection Characteristics

Infection sources were classified as abdominal, pulmonary, cutaneous, or urinary based on clinical, radiological, and microbiological data. Multiple concurrent infection sites were recorded when present. Microbiological data included:

Screening culture results: Surveillance cultures obtained at ICU admission or during ICU stay, categorized as carbapenem-resistant *Enterobacteriaceae* (CRE+), extended-spectrum beta-lactamase producing organisms (ESBL+), vancomycin-resistant *Enterococcus* (VRE+), methicillin-resistant *Staphylococcus aureus* (MRSA+), or negative.

Positive cultures: All organisms isolated from clinical specimens (blood, respiratory, urine, wound, intra-abdominal, etc.) during the infection episode. Infection complexity was systematically classified for each patient based on the number of distinct pathogens isolated and the number of anatomical sites involved. Patients were categorized as: (1) monomicrobial single-site: one pathogen isolated from one anatomical source; (2) monomicrobial multi-site: the same pathogen isolated from two or more distinct anatomical sources, suggesting systemic dissemination; or (3) polymicrobial: two or more distinct pathogens identified, regardless of the number of sites. Infection complexity category was included as a covariate in multivariable analysis. Species identification was performed using matrix-assisted laser desorption/ionization time-of-flight mass spectrometry (MALDI-TOF MS- MALDI Biotyper, Bruker Daltonics, Bremen, Germany) or conventional biochemical methods.

Antimicrobial resistance genes molecular characterization of resistance mechanisms was performed when available, including detection of carbapenemases (NDM, OXA-48, KPC, VIM, OXA-23, OXA-24), extended-spectrum beta-lactamases (CTX-M, SHV, TEM), and other resistance determinants (MEC-A, QNRS, aminoglycoside-modifying enzymes) using the Unyvero^®^ multiplex PCR-based syndromic testing system (Curetis GmbH, Holzgerlingen, Germany), according to the manufacturer’s instructions. It is important to note that multiplex PCR-based syndromic testing (Unyvero^®^) was performed as a complementary diagnostic tool alongside standard microbiological culture and antimicrobial susceptibility testing (AST) in all cases, and not as a replacement for conventional microbiology. Carbapenem resistance was phenotypically confirmed in all eligible isolates based on conventional culture results, with susceptibility profiles determined according to EUCAST breakpoints for meropenem and imipenem. Resistance gene identification via Unyvero^®^ PCR provided additional molecular characterization of the underlying resistance mechanisms but was not the sole criterion for MDR classification.

Antimicrobial susceptibility testing (AST): Eravacycline susceptibility was confirmed by AST in all 40 patients prior to treatment initiation, as this was a mandatory criterion for eravacycline use in our institution. Complete AST panels were available retrospectively for 25 of 40 patients (62.5%); full antibiogram data were not systematically archived electronically for the entire cohort. Full antimicrobial susceptibility profiles for the 25 isolates with available data are presented in Supplementary Table S4. No cases of primary bloodstream infection were identified in this cohort; all infections were of defined anatomical source. Source control was achieved in all patients who underwent surgical intervention.

#### 2.3.3. Treatment Characteristics

Eravacycline initiation was based on the following clinical criteria, applied on a case-by-case basis by the treating intensivist: (1) failure of at least two prior antimicrobial regimens, defined as persistent clinical and/or microbiological signs of infection despite adequate therapy; (2) microbiological confirmation of the causative pathogen with documented susceptibility to eravacycline on antimicrobial susceptibility testing (AST); and (3) availability of eravacycline as a salvage option, taking into account the limited formulary of last-resort agents at our institution (eravacycline, cefiderocol). No formal institutional protocol governed eravacycline initiation during the study period; decisions reflected individualized clinical judgment based on the above criteria.

All antimicrobial agents recorded as prior therapy represent sequential treatment attempts for the same microbiologically confirmed infectious episode that ultimately required salvage therapy with eravacycline. These agents targeted the same causative pathogen(s) and were discontinued due to clinical or microbiological treatment failure. Prior antibiotic exposures for unrelated infections during the ICU stay were not included.

Eravacycline administration data included:

- Start date and ICU day of initiation (calculated from ICU admission date);
- Treatment duration (days);
- Dosing regimen (standard dose: 1 mg/kg IV every 12 h);
- All concomitant antimicrobial agents administered during eravacycline therapy were recorded, including drug name, start and stop dates, and indication.

Eravacycline was initiated on the same day the MDR culture result with confirmed eravacycline susceptibility became available, with no delay between culture result and treatment initiation in all cases. The ICU day of eravacycline initiation therefore reflects the interval from ICU admission to availability of the MDR microbiological result.

Combination antimicrobial therapy was used in 28 patients (70.0%) and was driven by two distinct clinical indications: (1) the presence of Gram-positive co-isolates (Enterococcus faecium, MRSA) in polymicrobial infections (*n* = 6, 15.0%), requiring agents with dedicated Gram-positive activity (linezolid, vancomycin) alongside eravacycline; and (2) polymicrobial MDR Gram-negative infections involving multiple resistant pathogens (*n* = 21, 52.5%), where combination regimens ensured comprehensive microbiological coverage of all identified pathogens. Twelve patients (30.0%) received eravacycline as monotherapy.

#### 2.3.4. Laboratory Parameters

Serial inflammatory markers were collected at three time points:

- Initiation: Within 24 h before or after eravacycline start;
- During treatment: Mid-treatment assessment (day 3–5 of therapy);
- End of treatment: Within 48 h of eravacycline completion or discontinuation.

Laboratory parameters included:

- White blood cell count (WBC, cells/μL);
- C-reactive protein (CRP, mg/L);
- Procalcitonin (PCT, ng/mL).

Laboratory analyses were performed in the hospital’s central laboratory using standardized automated platforms.

### 2.4. Outcome Measures

#### 2.4.1. Primary Outcome

All-cause mortality during hospitalization (deceased vs. survived to discharge).

#### 2.4.2. Secondary Outcomes

1. Clinical outcome: Categorized as CURED (resolution of signs and symptoms of infection, no need for additional antimicrobial therapy) or DECEASED.
2. Microbiological eradication: Assessed systematically at the end of eravacycline treatment in all patients using a dual confirmation approach: (1) microbiological confirmation, defined as a negative follow-up culture from the index infection site at treatment completion; and (2) clinical improvement, defined as reduction in vasopressor requirements and improvement in respiratory parameters (decreased oxygen requirements and/or ventilatory support weaning). Eradication was classified as SUCCESS only when both microbiological and clinical criteria were simultaneously met. Patients without a documented negative follow-up culture were classified as FAILURE, regardless of clinical trajectory.
3. Cause of death: Categorized as INFECTION (death directly attributable to uncontrolled infection or septic shock) or OTHER CAUSES (including multi-organ failure, cardiovascular events, underlying disease progression, or withdrawal of care for non-infectious reasons).
4. ICU length of stay (days from ICU admission to ICU discharge or death).
5. Total hospitalization duration (days from hospital admission to discharge or death).

Outcomes were adjudicated by two independent reviewers (S.A.R.; A.B.) via medical record review; discrepancies resolved by a third (O.H.B.).

### 2.5. Statistical Analysis

#### 2.5.1. Descriptive Statistics

Continuous variables were assessed for normality using the Shapiro–Wilk test and visual inspection of Q-Q plots. Normally distributed variables are presented as mean ± standard deviation (SD), while non-normally distributed variables are presented as median with interquartile range [IQR]. Categorical variables are reported as frequencies and percentages.

#### 2.5.2. Comparative Analysis

Patients were stratified by primary outcome (survived vs. deceased) for univariate comparisons. For continuous variables, Student’s *t*-test was used for normally distributed data and Mann–Whitney U test for non-normally distributed data. For categorical variables, chi-square test or Fisher’s exact test (when expected cell counts < 5) were employed. Statistical significance was defined as two-tailed *p* < 0.05.

#### 2.5.3. Multivariable Analysis

Logistic regression was performed to explore variables associated with mortality.

Variable selection was based on clinical relevance and univariate significance (*p* < 0.10). The following candidate variables were considered for inclusion: (1) clinical characteristics—age, sex, weight, APACHE II score at ICU admission, SOFA score at ICU admission, ARDS, vasopressor use, mechanical ventilation duration, and surgical intervention; (2) infection characteristics—infection source, infection complexity category (monomicrobial single-site, monomicrobial multi-site, polymicrobial), and presence of specific carbapenemase genes; (3) treatment variables—ICU day of eravacycline initiation, treatment duration, and use of combination antimicrobial therapy; and (4) laboratory parameters—WBC, CRP, and PCT at treatment initiation, mid-treatment, and end of treatment. APACHE II and SOFA scores at ICU admission were used as proxies for baseline disease severity, as retrospective rescoring at infection onset or eravacycline initiation was not feasible given the retrospective study design. Infection complexity (monomicrobial single-site as reference, monomicrobial multi-site, and polymicrobial as binary indicators) was included a priori based on clinical rationale, regardless of univariate *p*-value. Continuous variables were standardized (z-score transformation) to enable comparison of effect sizes across different measurement scales. Results are presented as odds ratios (OR) with 95% confidence intervals. Model performance was assessed using accuracy, sensitivity, specificity, and area under the receiver operating characteristic curve (AUC-ROC). Multicollinearity was evaluated using variance inflation factors (VIF), with VIF > 5 indicating problematic collinearity.

#### 2.5.4. Missing Data

Missing data were handled as follows:

- For laboratory values: Missing values at specific timepoints were not imputed for descriptive analyses but were imputed using median values for machine learning analyses in order to preserve sample size and maintain model stability.
- For baseline characteristics: Patients with missing primary outcome data were excluded from analysis.
- The proportion of missing data for each variable is reported in the Supplementary Materials.
- Sensitivity analyses were performed excluding patients with more than 20% missing variables to assess the robustness of the findings.

### 2.6. Machine Learning Analysis

All machine learning analyses described below were performed exclusively as exploratory and hypothesis-generating tools. Given the sample size of *n* = 40 and the clinical heterogeneity of the cohort, no confirmatory or predictive conclusions are drawn from these analyses. Results should be interpreted solely as pattern-identification exercises to inform future prospective research.

#### 2.6.1. Feature Engineering and Preprocessing

A comprehensive feature matrix was constructed including:

1. Demographics: age, sex, weight.
2. Severity scores: APACHE II and SOFA at admission.
3. Clinical interventions: ARDS, vasopressor use, surgical procedures, mechanical ventilation duration.
4. Treatment variables: Eravacycline duration, ICU day of initiation, combined antibiotics.
5. Laboratory values: WBC, CRP, and PCT at all timepoints.
6. Infection characteristics: Infection sources (binary indicators), presence of specific resistance genes.

Categorical variables were encoded as binary indicators (one-hot encoding for infection sources and resistance genes). Missing values were imputed using median imputation for continuous variables. All features were standardized using z-score normalization (StandardScaler from scikit-learn) prior to model training.

#### 2.6.2. Unsupervised Clustering

Unsupervised clustering was performed to identify distinct patient phenotypes using K-means clustering algorithm. The optimal number of clusters was determined using:

1. Silhouette score: Measures cohesion within clusters and separation between clusters (range: −1 to +1, higher is better);
2. Davies–Bouldin index: Measures average similarity ratio of each cluster with its most similar cluster (lower is better);
3. Elbow method: Visual inspection of within-cluster sum of squares vs. number of clusters.

Clustering was performed on the standardized feature matrix. The number of clusters was evaluated from k = 2 to k = 5. Principal component analysis (PCA) was used for visualization, reducing dimensionality to 2 components while retaining >85% of variance.

For each identified cluster, we characterized:

- Patient demographics and baseline severity;
- Infection characteristics and resistance patterns;
- Treatment characteristics;
- Laboratory trajectories;
- Clinical outcomes.

Statistical comparisons between clusters were performed using Kruskal–Wallis test for continuous variables and chi-square test for categorical variables.

Given the relatively small sample size, clusters with very few observations (*n* = 1) were considered descriptive only and were not included in formal comparative analyses. The clustering approach was used to explore potential patterns of patient heterogeneity rather than to define reproducible phenotypes.

#### 2.6.3. Supervised Classification

Three machine learning models were developed to explore patterns associated with mortality:

1. Logistic Regression: Linear model with L2 regularization, maximum iterations = 1000.
2. Random Forest Classifier: Ensemble method with 100 decision trees, maximum depth = 5, minimum samples per leaf = 2.
3. Gradient Boosting Classifier: Ensemble method with 100 boosting stages, maximum depth = 3, learning rate = 0.1.

Model Training and Validation:

- Target variable: Binary mortality outcome (0 = survived, 1 = deceased);
- Cross-validation: 5-fold stratified cross-validation to maintain class balance across folds;
- Random state: Fixed at 42 for reproducibility;
- Performance metrics: Area under ROC curve (AUC), accuracy, sensitivity, specificity, precision, recall, F1-score.

Feature Importance: For tree-based models (Random Forest, Gradient Boosting), feature importance was calculated using Gini importance (mean decrease in impurity). For Logistic Regression, absolute coefficient values were used as importance measures. Features were ranked by importance, and the top 15 features were reported.

Model Interpretation:

- ROC curves were plotted for all models;
- Confusion matrices were generated using default decision threshold (0.5);
- Predicted probability distributions were analyzed separately for survivors and non-survivors;
- Cross-validation scores were reported as mean ± standard deviation across folds.

#### 2.6.4. Antibiotic Combination Analysis

For the most frequently used combinations of antibiotics (>5% of patients), we performed subgroup analyses comparing outcomes in patients receiving vs. not receiving each agent. This included:

- Mortality rates with vs. without the antibiotic;
- Microbiological eradication with vs. without the antibiotic;
- Mean baseline severity scores (APACHE II, SOFA) in patients receiving each antibiotic.

These analyses were descriptive; statistical testing was not performed due to small sample sizes and high risk of confounding by indication (sicker patients received broader coverage).

#### 2.6.5. Software and Reproducibility

All statistical analyses were performed using Python 3.12.12 with the following libraries:

- Data manipulation: pandas 2.2.2, numpy 2.0.2;
- Statistical analysis: scipy 1.16.2;
- Machine learning: scikit-learn 1.6.1;
- Visualization: matplotlib 3.10.0, seaborn 0.13.2.

All analysis code was version-controlled and is available upon reasonable request. Random seeds were set to ensure reproducibility of all machine learning analyses.

Given the limited sample size, there is a risk of model overfitting, particularly for more complex algorithms such as Random Forest and Gradient Boosting. Therefore, model performance metrics should be interpreted with caution.

### 2.7. Ethical Considerations

All data were de-identified prior to analysis. Patient confidentiality was maintained throughout the study. The study posed no additional risk to participants beyond standard clinical care.

### 2.8. Data Availability

The datasets generated and analyzed during the current study are not publicly available due to patient privacy regulations but are available from the corresponding author upon reasonable request and with appropriate institutional approvals.

AI tools (ChatGPT (GPT-4, OpenAI, San Francisco, CA, USA) were used solely for language editing and improvement of readability. No AI tools were used for data analysis, interpretation of results, or generation of scientific content. All scientific content and conclusions are the sole responsibility of the authors.

## 3. Results

### 3.1. Patient Characteristics

Between 1 February 2024 and 30 September 2025, 40 patients with microbiologically confirmed multidrug-resistant infections received eravacycline as salvage therapy in our ICU. The median age was 58 years (IQR: 47–69), and 57.5% were male. Patients presented with severe critical illness, with a mean APACHE II score of 16.4 ± 6.9 and SOFA score of 5.5 ± 3.3 at ICU admission. The majority required intensive organ support: 35 patients (87.5%) received vasopressor therapy, 30 patients (75%) required mechanical ventilation with a median duration of 25.2 days (IQR: 13–35), and 11 patients (27.5%) developed ARDS. The median ICU length of stay was 31.4 days (IQR: 16–44), with a total hospital stay of 41.0 days (IQR: 23–52).

### 3.2. Infection Characteristics and Microbiology

**Figure 2:**
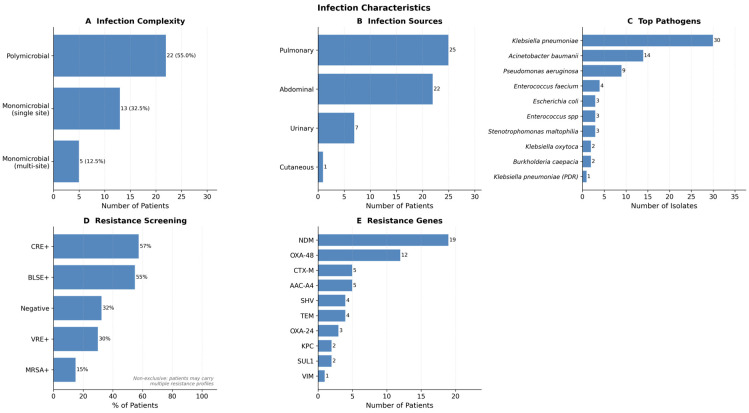
Infection Characteristics and Microbiology.

The predominant infection sources were pulmonary (62.5%) and intra-abdominal (55.0%). Infection complexity analysis revealed that 22 patients (55.0%) had polymicrobial infections involving two or more distinct pathogens, 13 patients (32.5%) had monomicrobial infections confined to a single anatomical site, and 5 patients (12.5%) had monomicrobial infections with isolation of the same pathogen from multiple anatomical sites, consistent with systemic dissemination. The latter group comprised exclusively *Klebsiella pneumoniae* (4 patients) and *Burkholderia cepacia* (1 patient). Among polymicrobial cases, the median number of distinct pathogens per patient was 2 (range 2–7), with patient M.I. representing the most complex case, with 7 distinct species isolated from two sites. These findings underscore the high microbiological complexity of the cohort and are represented in Figure 2. Screening cultures taken at admittance in ICU revealed extensive antimicrobial resistance: 23 patients (57.5%) were positive for carbapenem-resistant *Enterobacteriaceae* (CRE), 22 patients (55.0%) for extended-spectrum beta-lactamase producing organisms (ESBL+), 12 patients (30.0%) for vancomycin-resistant *Enterococcus* (VRE+), and 6 patients (15.0%) for methicillin-resistant *Staphylococcus aureus* (MRSA+).

*Klebsiella pneumoniae* was the most frequently isolated pathogen (30 patients, 75.0%), followed by *Acinetobacter baumannii* (14 patients, 35.0%) and *Pseudomonas aeruginosa* (9 patients, 22.5%). Given the intrinsic lack of activity of eravacycline against *Pseudomonas aeruginosa*, these cases were managed either with combination antimicrobial therapy including agents with established anti-pseudomonal activity or were considered as co-isolates in polymicrobial infections not primarily targeted by eravacycline therapy. All 9 patients with *Pseudomonas aeruginosa* isolates presented in the context of polymicrobial infections; none had *Pseudomonas aeruginosa* as the sole causative pathogen. In 4 of 9 patients, at least one agent with established anti-pseudomonal activity was co-administered: ceftolozane-tazobactam (1 patient), colistin (2 patients), meropenem (2 patients), and amikacin (1 patient), with some patients receiving more than one agent. In 2 further patients (M.I., M.M.M.), eravacycline was initiated on ICU day 9 and day 14 respectively, with death occurring within 2 days of initiation, suggesting that rapid clinical deterioration precluded therapeutic escalation.

Molecular characterization of resistance mechanisms revealed a high prevalence of carbapenemase genes, with NDM detected in 19 isolates (47.5%) and OXA-48 in 12 isolates (30.0%). Additional resistance determinants included CTX-M (12.5%), KPC (5.0%), and VIM (2.5%) (Figure 2).

### 3.3. Treatment Characteristics

Eravacycline was initiated at a median of ICU day 9 (IQR 4–28, range 2–81), reflecting the interval from ICU admission to availability of the MDR microbiological result with confirmed eravacycline susceptibility, as treatment was initiated on the same day the culture result became available in all cases. Twelve patients (30.8%) received eravacycline within the first 5 ICU days, 12 patients (30.8%) between days 6–14, 8 patients (20.5%) between days 15–30, and 7 patients (17.9%) after day 30. The median treatment duration was 7.0 days (IQR: 5.8–10.2). Most patients received combination antimicrobial therapy, with colistin (22.5%), linezolid (17.5%), and meropenem (15.0%) being the most frequently co-administered agents (Table 1).

### 3.4. Laboratory Trends

**Figure 3:**
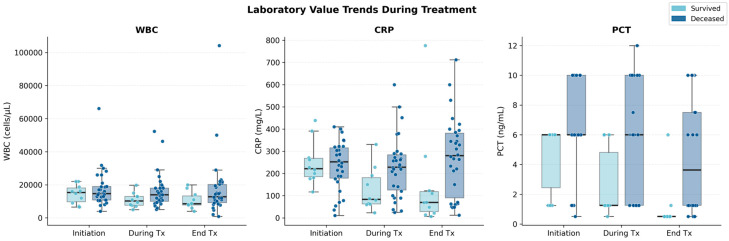
Evolution of inflammatory markers (WBC, CRP, and procalcitonin) during eravacycline therapy, including values at treatment initiation, during therapy, and at treatment completion.

Inflammatory markers at treatment initiation showed marked elevation: median white blood cell count 14,700 cells/μL (IQR: 10,750–18,850), C-reactive protein 242 mg/L (IQR: 180–308.5), and procalcitonin 6.0 ng/mL (IQR: 6.0–10.0). During the course of treatment, procalcitonin demonstrated the most substantial decline (median end-of-treatment value: 1.25 ng/mL, IQR: 0.5–6.38), while C-reactive protein showed variable responses (median end-of-treatment: 248 mg/L, IQR: 63.8–351.2) and white blood cell counts remained relatively unchanged (median: 12,000 cells/μL, IQR: 8250–18,750) (Figure 3).

### 3.5. Clinical Outcomes

**Figure 4:**
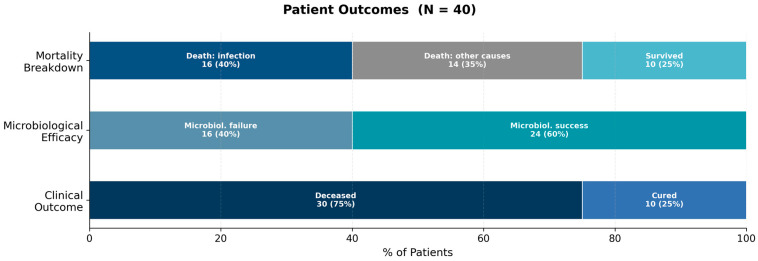
Patient outcomes in the study cohort (N = 40), presented across three complementary panels. The bottom panel (Clinical Outcome) shows the overall distribution of patients by final outcome: 30 patients (75%) died during hospitalization and 10 patients (25%) were discharged with clinical cure. The middle panel (Microbiological Efficacy) shows the proportion achieving microbiological success (eradication of the index pathogen confirmed by negative follow-up cultures): 24 patients (60%) versus microbiological failure in 16 patients (40%). The top panel (Mortality Breakdown) further stratifies the 30 deceased patients by attributed cause of death: 16 patients (40% of the total cohort) died due to refractory infection, while 14 patients (35%) died of other causes (multiorgan failure, haemorrhic complications, or non-infectious aetiology). Note that the top panel subdivides only the deceased patients from the bottom panel—the 10 surviving patients are shown for reference but are not further subdivided.

Overall mortality was 75% (30/40 patients), with only 10 patients (25%) achieving clinical cure. Microbiological eradication, defined as eradication or presumed eradication of the causative pathogen, was documented in 24 patients (60%). Among the 30 deaths, 16 (53.3%) were attributed to an uncontrolled source of infection, while 14 (46.7%) resulted from other causes including multi-organ failure, cardiovascular collapse, and underlying comorbidities (Figure 4).

Among the 9 patients with *Pseudomonas aeruginosa* co-infection, all 9 (100%) died during hospitalization, compared to 21 of 31 patients (67.7%) without *Pseudomonas aeruginosa* isolation. Given the small subgroup size and the confounding effect of greater overall infection complexity in polymicrobial cases, no formal statistical comparison was performed; these figures are reported descriptively.

### 3.6. Predictors of Mortality

Univariate analysis identified several factors associated with mortality (Table 2). Baseline disease severity, as measured by APACHE II score, was significantly higher in non-survivors (median 19.0, IQR: 14.2–22.5) compared to survivors (median 10.5, IQR: 7.5–13.5; *p* = 0.0024). Age showed a trend toward higher values in non-survivors (median 64.5 vs. 47.0 years; *p* = 0.0566), although this did not reach statistical significance.

Resolution of inflammatory markers during treatment was strongly associated with survival (Figure 2). Patients who survived demonstrated significantly lower C-reactive protein levels during treatment (median 83.5 vs. 228.0 mg/L, *p* = 0.0235) and at treatment completion (median 69.5 vs. 280.0 mg/L, *p* = 0.0299). Procalcitonin showed consistent associations across timepoints, with significant differences at initiation (*p* = 0.0328), during treatment (*p* = 0.0204), and most prominently at the end of treatment. At completion, survivors achieved near-normalization (median 0.5 ng/mL) compared to non-survivors who maintained elevated levels (median 3.6 ng/mL, *p* = 0.0006).

Notably, neither the timing of eravacycline initiation (*p* = 1.0) nor treatment duration (*p* = 0.25) demonstrated a statistically significant association with mortality. Other variables, including SOFA score (*p* = 0.098), vasopressor use (*p* = 0.089), and leukocyte counts during and at the end of treatment (*p* = 0.080 and *p* = 0.098, respectively), showed trends toward significance but did not reach statistical thresholds. Weight, sex, mechanical ventilation duration, presence of ARDS, and surgical interventions were not associated with mortality.

Multivariable logistic regression analysis identified APACHE II score as the strongest predictor of mortality in this cohort (OR 2.70 per standardized unit increase), followed by vasopressor use (OR 1.74), SOFA score (OR 1.67), ICU day of eravacycline initiation (OR 1.66), and age (OR 1.28). Infection complexity, modeled as a composite binary variable, showed that polymicrobial infection was associated with increased odds of mortality (OR 1.46), reflecting the impact of overall microbial burden rather than infection distribution patterns. Eravacycline duration was associated with a modest reduction in mortality odds (OR 0.90), consistent with the interpretation that longer treatment reflects a more favorable clinical trajectory rather than a direct therapeutic effect. ARDS demonstrated only a minimal association with outcome (OR 1.20).

Model accuracy was 82.5%. All continuous variables were standardized prior to analysis; odds ratios therefore reflect the effect of a one standard deviation increase in each variable.

Bootstrap internal validation (B = 1000) yielded an optimism-corrected AUROC of 0.776 (95% CI 0.643–0.900), with mean optimism of 0.097 and 0.632+ estimate of 0.738, indicating moderate discriminative ability after adjustment for overfitting (Supplementary Figure S1, Supplementary Tables S2 and S3). All continuous variables were standardized prior to analysis; odds ratios reflect the effect of a one standard deviation increase.

### 3.7. Cluster Analysis

The following cluster and machine learning analyses are exploratory in nature and should be interpreted with caution given the limited sample size (*n* = 40) and population heterogeneity. No confirmatory or predictive conclusions are drawn from these results.

Unsupervised machine learning identified five distinct patient phenotypes with markedly different clinical trajectories (Table 3).

Cluster 0 (*n* = 12) represented the most critically ill patients with the highest disease severity (mean APACHE II 22.0, SOFA 8.8). All patients in this cluster received vasopressor support, 91.7% underwent surgical intervention, and all required mechanical ventilation. This group experienced 100% mortality despite aggressive intervention, with the shortest ICU stay (mean 15.8 days), suggesting rapid progression to death. Microbiological eradication was achieved in only 16.7% of cases. Inflammatory markers remained severely elevated throughout treatment (end-of-treatment CRP 321.5 mg/L, PCT 6.54 ng/mL).

Cluster 1 (*n* = 12) comprised younger patients (mean age 49.4 years) with the lowest disease severity (mean APACHE II 11.7, SOFA 3.6). Despite having the highest burden of resistance genes (mean 3.3 per patient), this group achieved the best outcomes, with 58.3% mortality (41.7% survival) and 75% microbiological eradication. Inflammatory markers demonstrated favorable trajectories, with procalcitonin declining from 5.42 to 3.31 ng/mL. This cluster had an intermediate ICU length of stay (25.6 days).

Cluster 2 (*n* = 14) consisted of the oldest patients (mean age 66.9 years) with moderate disease severity (APACHE II 15.8, SOFA 4.2). This group demonstrated the longest ICU stays (mean 40.3 days) and hospital durations (47.5 days), suggesting prolonged critical illness. Notably, this cluster achieved the highest microbiological eradication (85.7%), with excellent inflammatory marker resolution (PCT declined from 5.84 to 1.66 ng/mL), yet 64.3% of patients still died. Eravacycline was initiated latest in this group (mean ICU day 23.3).

Clusters 3 and 4 each contained single patients with unique clinical profiles and were therefore considered descriptive only and excluded from comparative statistical analyses.

### 3.8. Machine Learning Prediction of Mortality

Random Forest analysis suggested that procalcitonin at treatment completion emerged as the variable most strongly associated with mortality in this exploratory model (feature importance 1.11), followed by baseline APACHE II score (0.82), presence of brain surgery (0.67), age (0.67), and duration of mechanical ventilation (0.66). The model achieved a cross-validated area under the receiver operating characteristic curve of 0.767 ± 0.162 (training AUC of 1.0). However, the perfect training performance suggests potential overfitting, and these results should therefore be interpreted with caution.

**Figure 5:**
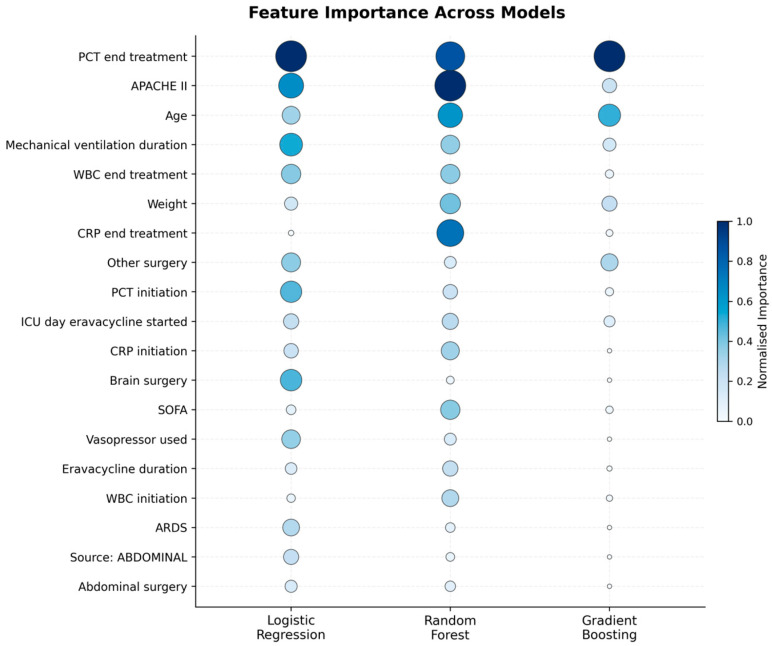
Machine Learning Prediction of Mortality.

In contrast, treatment-related variables demonstrated limited predictive value in this exploratory model: eravacycline timing (feature importance 0.33) and duration (0.25) ranked among the least important features. Similarly, baseline SOFA score (0.04) and C-reactive protein at treatment completion (0.04) contributed negligibly to mortality prediction (Figure 5).

(Logistic Regression AUC: 0.767 ± 0.178 (training AUC 0.997), Gradient Boosting AUC: 0.600 ± 0.178 (training AUC 1).)

Bootstrap internal validation (B = 1000) was performed to assess model optimism (Supplementary Table S1). Apparent AUC was 1.00 for all three models, reflecting expected overfitting on training data given the sample size and feature dimensionality. Mean optimism was 0.078 for logistic regression, 0.039 for random forest, and 0.099 for gradient boosting. Optimism-corrected AUCs were 0.922 (95% CI: 0.820–0.987), 0.961 (95% CI: 0.878–1.000), and 0.901 (95% CI: 0.723–0.997) respectively, indicating that discriminative performance remained high after correction. The 0.632 estimator, which provides a more conservative bias-corrected estimate, yielded AUCs of 0.835, 0.854, and 0.795. These findings suggest genuine discriminative signal beyond overfitting, though external validation in an independent cohort remains necessary.

### 3.9. Antibiotic Combination Therapy

Analysis of commonly co-administered antibiotics did not suggest any clear mortality benefit for a specific combination (Table 4). Patients receiving colistin demonstrated numerically lower mortality (66.7% vs. 77.4% without colistin), though this difference was not statistically significant. Notably, patients receiving aztreonam (100% mortality, mean APACHE II 22), metronidazole (100% mortality, mean APACHE II 25), or meropenem (83.3% mortality, mean APACHE II 17.8) had substantially higher baseline disease severity, likely reflecting selection bias where sicker patients received broader antimicrobial coverage.

## 4. Discussion

### 4.1. Key Findings

This retrospective cohort study evaluated outcomes in critically ill patients with multidrug-resistant infections treated with eravacycline as salvage therapy. Our findings demonstrate that despite achieving microbiological eradication in 60% of cases, overall mortality remained high at 75%. The median APACHE II score at ICU admission was 17 (IQR 11–21), which would conventionally predict a mortality of approximately 25–30%. The observed mortality of 75% substantially exceeds this prediction, likely reflecting the highly selected nature of the cohort—patients who had already failed a median of 4 prior antimicrobial regimens and were initiated on eravacycline as a last-resort salvage intervention. By the time salvage therapy was commenced, many patients had developed progressive organ dysfunction not fully captured by admission severity scores calculated days to weeks earlier. Importantly, survival appeared to be more closely related to baseline disease severity and the host ability to resolve systemic inflammation, rather than antimicrobial characteristics including timing or duration of eravacycline therapy.

### 4.2. Microbiological Eradication vs. Clinical Cure

A striking finding of our study is the dissociation between microbiological and clinical success. While 60% of patients achieved microbiological eradication or presumed eradication, only 25% survived to hospital discharge. This discordance was most evident in Cluster 2, where 85.7% microbiological eradication was accompanied by 64.3% mortality. These older patients (mean age 67 years) demonstrated excellent pathogen clearance and inflammatory marker resolution (PCT normalization), yet succumbed to complications of prolonged critical illness. This phenomenon has been increasingly recognized in the critical care literature, where antimicrobial therapy, though necessary, is insufficient to reverse established multi-organ dysfunction in severely ill patients [7,8,9,10].

Nearly half of all deaths (46.7%) were attributed to non-infectious causes, suggesting that once multi-organ failure is established, controlling the microbial pathogen alone may be insufficient to reverse clinical outcomes. This observation aligns with contemporary understanding of sepsis pathophysiology, where dysregulated host response and immunoparalysis contribute more to adverse outcomes than the causative organism itself [10,11,12].

### 4.3. Disease Severity Supersedes Antimicrobial Selection

Univariate and multivariable analyses consistently showed that baseline APACHE II score was strongly associated with mortality (*p* = 0.0024, OR 2.68). Survivors presented with substantially lower disease severity (APACHE II 10.5) compared to non-survivors (APACHE II 19.0). This 8.5-point difference translates to an approximately 30–40% difference in predicted mortality based on established APACHE II scoring systems [13,14,15,16,17].

The observed 75% mortality rate substantially exceeds the 25–30% mortality predicted by APACHE II and SOFA scores alone. This elevated mortality likely reflects the compounding effects of multidrug resistance, delayed appropriate therapy, and the salvage nature of eravacycline use in our cohort. Previous studies of carbapenem-resistant infections have reported mortality rates ranging from 40–70% [18,19], with rates exceeding 70% when treatment options are severely limited [20,21,22]. Our findings are consistent with the upper end of this range, suggesting that even with eravacycline availability, outcomes in extremely resistant infections remain poor.

### 4.4. Inflammatory Marker Trajectories as Prognostic Indicators

In the exploratory machine learning analysis, procalcitonin at treatment completion showed the strongest potential association with mortality (feature importance 1.11, *p* = 0.0006). Survivors achieved near-complete PCT normalization (0.5 ng/mL), while non-survivors maintained significantly elevated levels (3.6 ng/mL). This finding corroborates extensive literature supporting PCT-guided prognostication in sepsis and suggests that inability to resolve systemic inflammation, regardless of microbiological clearance, portends poor outcomes [13,23,24].

Interestingly, C-reactive protein, despite its widespread use as an inflammatory marker, demonstrated minimal predictive value (feature importance 0.04), possibly due to its longer half-life and non-specific elevations in critical illness. This observation supports the potential utility of procalcitonin for monitoring treatment response in severe infections [25,26,27].

### 4.5. Timing and Duration of Eravacycline

Perhaps our most clinically relevant negative finding is the absence of a statistically significant association between eravacycline initiation timing (*p* = 1.0, feature importance 0.33) or treatment duration (*p* = 0.25, feature importance 0.25) and mortality. Eravacycline was initiated at a median of 9 ICU days, clearly representing salvage therapy following failure of first-line agents. Both survivors and non-survivors received eravacycline at similar timepoints, suggesting that by the time multidrug-resistant organisms are identified and eravacycline is initiated, the trajectory toward survival or death may already be influenced by earlier disease progression.

This finding has important implications for antibiotic stewardship and clinical decision-making. While early appropriate antimicrobial therapy is a cornerstone of sepsis management [20,23,28], our data suggest that in the salvage setting—after failure of multiple prior antibiotics—the specific salvage agent and its timing may have limited observable impact on outcomes in this setting. This underscores the critical importance of empiric therapy selection, de-escalation based on culture results, and early source control, as once patients enter the salvage therapy phase, outcomes appear to be predominantly related to underlying physiology rather than antimicrobial choice.

### 4.6. Patient Phenotypes and Personalized Prognosis

Our unsupervised clustering analysis suggested the presence of three potential clinical phenotypes with distinct outcomes.

Cluster 0, representing a high-severity phenotype, experienced universal mortality despite maximal support. Recognizing this phenotype early—characterized by APACHE II > 20, SOFA > 8, universal vasopressor dependence, and absent inflammatory marker improvement—may provide insights relevant to goals-of-care discussions.

Cluster 1, the lower-severity phenotype, had favorable baseline characteristics and achieved reasonable outcomes despite carrying the highest resistance burden. This paradox suggests that in patients with adequate physiologic reserve, even extensively resistant organisms can be successfully treated.

Cluster 2, the prolonged critical illness phenotype, presents the greatest clinical conundrum: achieving excellent microbiological control yet succumbing to complications of prolonged critical illness. These elderly patients with extended ICU stays (40 days) represent a growing population in modern intensive care, where life-sustaining technology can prolong survival beyond the body’s capacity for recovery [28,29,30].

The clustering results should be interpreted with caution, as the relatively small sample size and the presence of very small clusters limit the reproducibility and generalizability of the identified phenotypes. Therefore, these findings should be considered exploratory and hypothesis-generating rather than definitive.

### 4.7. Comparison to Published Literature

Limited published data exist on eravacycline use in critically ill patients, as most clinical trials excluded severely ill ICU populations [31]. The IGNITE trials reported clinical cure rates of 86–92% for complicated intra-abdominal infections, substantially higher than our 25% cure rate. However, those trials enrolled predominantly non-ICU patients with lower rates of organ dysfunction and excluded patients with APACHE II >30 or those requiring vasopressor support [32,33]. Our cohort, with 87.5% vasopressor use and mean APACHE II 16.4, represents a far more critically ill population.

More recently, a large multicenter real-world study published in 2026 evaluated eravacycline use in 1796 patients with *Acinetobacter baumannii* and *Klebsiella pneumoniae* infections [33]. The authors reported high susceptibility rates (96% overall and approximately 98% among carbapenem-resistant isolates), along with favorable clinical outcomes, including a treatment success rate of 82.6% at the end of therapy and 83.57% clinical cure at day 30. Importantly, outcomes were not significantly influenced by the use of monotherapy versus combination therapy. However, predictors of treatment failure included elevated inflammatory markers, bloodstream infection, sepsis, and invasive procedures.

In contrast to these findings, our cohort demonstrated substantially lower clinical cure rates and higher mortality. Notably, the majority of patients in that cohort were not critically ill to the same extent as those in our ICU population. This may be explained by the more severe baseline condition of our patients, the high proportion requiring vasopressor support, and the frequent use of eravacycline as late salvage therapy after multiple prior antibiotic failures. These differences highlight the critical impact of host factors and disease severity on clinical outcomes in critically ill populations.

All *Pseudomonas aeruginosa* isolates in our cohort occurred within polymicrobial infections. In 4 of 9 affected patients, dedicated anti-pseudomonal therapy was co-administered, including ceftolozane-tazobactam, colistin, meropenem, and amikacin in various combinations. In 2 patients, rapid deterioration within 48 h of eravacycline initiation precluded therapeutic escalation. However, in 2 patients with prolonged ICU courses, the absence of documented anti-pseudomonal coverage represents a treatment gap that we acknowledge as a limitation of this real-world cohort. This heterogeneity reflects the inherent complexity of prescribing decisions in critically ill patients with polymicrobial infections, where the relative contribution of individual co-isolates to the infectious syndrome is not always clinically apparent. Descriptively, mortality among all 9 patients with *Pseudomonas aeruginosa* co-isolation was 100%, compared to 67.7% in those without, though this difference likely reflects greater overall infection complexity rather than the effect of anti-pseudomonal coverage decisions alone.

Real-world evidence from other salvage antibiotics for MDR infections provides more appropriate context. Studies of ceftazidime-avibactam for CRE infections reported mortality rates of 32–50% [22,34,35], while colistin-based regimens have demonstrated mortality rates of 35–60% [36,37,38]. Our 75% mortality rate, while higher, likely reflects the more severe baseline illness in our cohort, extensive prior antibiotic failures, and the salvage nature of eravacycline use. A significant proportion of our patients had already failed multiple last-resort agents before receiving eravacycline.

### 4.8. Limitations

This study has several important limitations that must be considered when interpreting our findings. First and most critically, the single-arm observational design without a control group prevents any causal inference regarding eravacycline eradication. We cannot determine whether outcomes would have been better, worse, or unchanged with alternative antimicrobial regimens. The high mortality rate may reflect disease severity, limitations of salvage therapy in general, specific inadequacies of eravacycline, or a combination of these factors.

Second, selection bias is inherent to this retrospective cohort. Eravacycline was chosen for patients who had failed multiple prior antibiotics, creating a population enriched for treatment-refractory infections and severe illness. This limits generalizability to earlier-line therapy or less severely ill patients.

Third, the relatively small sample size (*n* = 40) limits statistical power for detecting modest treatment effects and subgroup differences. Several variables showed trends toward significance (SOFA, vasopressor use, WBC trends) that might reach statistical significance in larger cohorts. The clustering analysis, while revealing clinically meaningful phenotypes, should be validated in independent datasets. Moreover, the small number of patients in the monomicrobial multi-site subgroup (*n* = 5) precludes reliable estimation of the associated mortality risk, and the corresponding odds ratio should be interpreted with particular caution. Also, the limited sample size (*n* = 40) relative to the number of candidate features results in a high risk of overfitting, particularly for complex models such as random forest and gradient boosting. Bootstrap internal validation with 1000 resamples demonstrated modest optimism (3.9–9.9%) and corrected AUCs of 0.90–0.96, suggesting retained discriminative ability after adjustment; however, these estimates derive from internal validation only and require confirmation in external cohorts before any clinical application.

Fourth, we could not account for all potential confounders including adequacy of source control, timing and appropriateness of initial empiric therapy, specific supportive care practices, or unmeasured patient factors (frailty, functional status, goals of care). These factors likely influenced outcomes but were not systematically captured.

Fifth, structured comorbidity data were not systematically captured in the pharmacy registry used as the primary data source, and retrospective extraction from individual patient records was not feasible for all 40 cases. The absence of a comprehensive comorbidity profile (e.g., Charlson Comorbidity Index) represents a limitation of the present study and may have prevented full adjustment for underlying disease burden in the multivariable analyses.

Sixth, microbiological eradication assessment was based on clinical and microbiological criteria available in routine practice, not protocol-specified microbiological evaluations. Some patients had insufficient follow-up cultures to definitively document eradication. Additionally, the definition of clinical cure in critically ill patients is inherently subjective and influenced by complex decisions around withdrawal of life support. Anti-pseudomonal coverage was not consistently documented across all patients with *Pseudomonas aeruginosa* co-isolation. While most cases were explained by dedicated anti-pseudomonal co-therapy or rapid clinical deterioration, two patients with prolonged ICU courses had no documented anti-pseudomonal agent, representing a genuine treatment gap that cannot be retrospectively justified. This limits the conclusions that can be drawn regarding the adequacy of coverage in this subgroup and may have influenced outcomes independently of eravacycline therapy.

Seventh, the retrospective design precluded the systematic capture of exact dates of in-hospital death, limiting our ability to report a standardized day-30 mortality endpoint. Using total hospitalization duration as a surrogate, 17 patients (42.5%) died within 30 days of admission, while an additional 12 patients (30.0%) died during a hospitalization exceeding 30 days, leaving their day-30 survival status undetermined. Future prospective studies should incorporate pre-specified 30-day and 90-day mortality endpoints to enable more meaningful comparisons with published literature.

Furthermore, APACHE II and SOFA scores were available only at ICU admission; rescoring at infection onset or at the time of eravacycline initiation was not feasible retrospectively, as the complete physiological data required for recalculation at these timepoints were not systematically archived in the electronic records. Additionally, complete AST panels were available for only 25 of 40 patients (62.5%), as full antibiogram data were not systematically archived electronically for the entire cohort.

Finally, this represents a single-center experience in a specialized ICU, which may not reflect practices or populations in other settings. Our ICU cares for a high proportion of surgical patients with intra-abdominal infections, which may differ from medical ICUs with predominantly respiratory or bloodstream infections.

### 4.9. Clinical Implications and Future Directions

Our findings carry several directly actionable implications for ICU practice. Microbiological eradication, while necessary, is insufficient for survival in critically ill patients with MDR infections—supportive care quality, source control, and early appropriate empiric therapy remain the primary determinants of outcome. Once patients enter the salvage therapy phase after failing multiple antimicrobial regimens, the choice of salvage agent appears less impactful than underlying host physiology, underscoring the priority of infection prevention, antimicrobial stewardship, and optimization of initial therapy over late salvage strategies.

Inflammatory marker trajectories—particularly failure to achieve procalcitonin normalization by mid-treatment—may serve as early indicators of futility and should be incorporated into prognostic discussions and goals-of-care decisions. Similarly, early recognition of the highest-severity patient phenotype may facilitate timely transition to comfort-focused care when appropriate.

Future research should prioritize randomized controlled trials comparing eravacycline with other salvage regimens in well-defined critically ill populations, pharmacokinetic/pharmacodynamic studies to optimize dosing in patients with augmented renal clearance or on renal replacement therapy, and investigation of eravacycline as an earlier-line therapy before multiple treatment failures occur. External validation of the patient phenotypes identified in this exploratory analysis is necessary before prospective risk stratification can be implemented.

## 5. Conclusions

In this cohort of critically ill patients with multidrug-resistant Gram-negative infections requiring salvage therapy with eravacycline, moderate microbiological eradication was observed, while overall mortality remained high. Outcomes appeared to be more closely related to baseline disease severity and the resolution of systemic inflammation than to antimicrobial timing or duration. Antibiotherapy was frequently administered off-label, as many patients presented with multi-site infections complicated by septic shock.

The observed dissociation between microbiological response and survival highlights the complex interplay between pathogen burden and host response in advanced critical illness. These findings should be interpreted as exploratory, given the limited sample size, and require confirmation in larger prospective studies.

Overall, the results emphasize the importance of early appropriate therapy and optimal supportive care, as late salvage interventions may have limited impact once severe organ dysfunction is established.

## Figures and Tables

**Table 1:** Baseline characteristics, ICU admission diagnoses, and prior antimicrobial therapy of the study population (*n* = 40).

Characteristic	Value
Eravacycline duration (days), median [IQR]	7.0 [5.8–10.2]
ICU day of eravacycline initiation, median [IQR]	9.0 [4.8–26.5]
Concomitant antibiotics, *n* (%)	
Antibiotic	Patients, *n* (%)
Colistin	9 (22.5%)
Linezolid	7 (17.5%)
Meropenem	6 (15.0%)
Vancomycin	4 (10.0%)
Aztreonam	3 (7.5%)
Amikacin	2 (5.0%)
Metronidazole	2 (5.0%)
Amoxicillin/clavulanate	1 (2.5%)
Ampicillin/sulbactam	1 (2.5%)
Azithromycin ICU admission diagnostics	1 (2.5%)
Septic shock—abdominal origin	20 (50.0%)
Acute respiratory failure/VAP	9 (22.5%)
Neurological emergency (haemorrhage, myelitis)	3 (7.5%)
Polytrauma	2 (5.0%)
Septic shock—urinary origin	1 (2.5%)
Septic shock—pulmonary origin	1 (2.5%)
Mixed shock (hypovolaemic + septic)	1 (2.5%)
Other (COPD exacerbation, corrosive ingestion, aneurysm, cholecystectomy)	4 (10.0%)
Antimicrobial agents prior to eravacycline	
No prior antimicrobial therapy	2 (5.0%)
Carbapenems (meropenem, imipenem)	26 (65.0%)
Aminoglycosides (amikacin, gentamicin)	24 (60.0%)
Colistin	17 (42.5%)
Tigecycline	16 (40.0%)
Piperacillin/tazobactam	16 (40.0%)
Ceftazidime/avibactam	13 (32.5%)
Linezolid	13 (32.5%)
Vancomycin	9 (22.5%)
Imipenem/relebactam (Recarbrio)	6 (15.0%)
Aztreonam	4 (10.0%)
Fluoroquinolones (ciprofloxacin, moxifloxacin)	3 (7.5%)
Cefiderocol	2 (5.0%)
Ceftolozane/tazobactam (Zerbaxa)	2 (5.0%)
Infection source	
Abdominal	21 (52.5%)
Pulmonary	15 (37.5%)
Urinary	2 (5.0%)
Cutaneos	1 (2.5%)
Mechanical ventilation	30 (75.0%)
Vasopressor support	35 (87.5%)
ARDS	11 (27.5%)
Surgical intervention	29 (72.5%)
Source control achieved	29 (72.5%)
Primary bloodstream infection	0 (0.0%)
Eravacycline susceptibility confirmed by AST	40 (100.0%)
Complete AST panel available	25 (62.5%)

Abbreviations: ICU, intensive care unit; VAP, ventilator-associated pneumonia; COPD, chronic obstructive pulmonary disease; ARDS, acute respiratory distress syndrome; AST, antimicrobial susceptibility testing. Percentages are calculated from the total cohort (*n* = 40). Antibiotic categories are not mutually exclusive—individual patients may have received agents from multiple classes. Infection source categories are not mutually exclusive—patients with multiple concurrent infection sites were counted in each applicable category.

**Table 2:** Factors associated with mortality.

Variable	Survivors (*n* = 10)	Non-Survivors (*n* = 30)	*p*-Value
Procalcitonin at end of treatment (ng/mL)	0.5 [0.5–0.5]	3.6 [1.2–7.5]	0.0006 ***
APACHE II at admission (points)	10.5 [7.5–13.5]	19.0 [14.2–22.5]	0.0024 **
CRP_during_treatment (mg/L)	83.5 [62.8–180.8]	228.0 [125.2–284.0]	0.0235 *
CRP at end of treatment (mg/L)	69.5 [28.5–119.0]	280.0 [90.0–381.2]	0.0299 *
Procalcitonin at initiation (ng/mL)	6.0 [2.4–6.0]	6.0 [6.0–10.0]	0.0328 *
Procalcitonin during treatment (ng/mL)	1.2 [1.2–4.8]	6.0 [1.2–10.0]	0.0204 *
Age (years)	47.0 [29.0–62.0]	64.5 [51.2–74.0]	0.0566
SOFA at admission (points)	3.5 [2.2–4.8]	5.5 [3.0–9.0]	0.0984
Vasopressor use, *n* (%)	7 (70.0%)	28 (93.3%)	0.0893

Data are presented as median [interquartile range] unless otherwise specified. Categorical variables were analyzed using contingency tables; * *p* < 0.05, ** *p* < 0.01, *** *p* < 0.001. CRP: C-reactive protein; SOFA: Sequential Organ Failure Assessment; APACHE II: Acute Physiology and Chronic Health Evaluation II.

**Table 3:** Cluster analysis of patient phenotypes.

Cluster	*n*	Age (Years)	APACHE II	SOFA	ICU LOS (Days)	Mortality (%)	Eradication (%)
0	12	59.2	22.0	8.8	15.8	100.0	16.7
1	12	49.4	11.7	3.6	25	58.3	75.0
2	14	66.9	15.8	4.2	40.3	64.3	85.7

Data are presented as mean values. Clusters 3 and 4 (*n* = 1 each) were excluded from comparative analysis and are described in the text. Abbreviations: ICU, intensive care unit; LOS, length of stay.

**Table 4:** Descriptive analysis of the most frequently used concomitant antibiotics during eravacycline therapy.

Antibiotic	Patients *n* (%)	Mortality with (%)	Mortality Without (%)	Eradication with (%)	Eradication Without (%)	APACHE II	SOFA
Colistin	9 (22.5%)	66.7	77.4	77.8	54.8	14	5
Linezolid	7 (17.5%)	57.1	78.8	57.1	60.6	15	5
Meropenem	6 (15.0%)	83.3	73.5	50	61.8	17	5
Vancomycin	4 (10.0%)	75	75	50	61.1	14	3
Aztreonam	3 (7.5%)	100	73	66.7	59.5	22	6
Amikacin	2 (5.0%)	100	73.7	50	60.5	16	5
Metronidazole	2 (5.0%)	100	73.7	50	60.5	25	9

APACHE II and SOFA values are presented as means. These analyses are descriptive only; no formal statistical comparisons were performed because of small subgroup sizes and likely confounding by indication.

## Data Availability

The data supporting the findings of this study are available within the article. Additional data are available from the corresponding author upon reasonable request.

## Supplementary Materials

The following supporting information can be downloaded at: https://www.mdpi.com/article/10.3390/jcm15145631/s1, Table S1: Bootstrap optimism-corrected internal validation summary for all three machine learning models (Logistic Regression, Random Forest, Gradient Boosting; B = 1000, *n* = 40), including apparent AUROC, mean optimism, and corrected AUROC; Table S2: Performance table for the 8-feature logistic regression model, including bootstrap-corrected Brier score, calibration slope, and calibration intercept; Table S3: Odds ratio table for the 8-feature logistic regression model; Table S4: Full antimicrobial susceptibility profiles for 25 isolates with available data; Figure S1: Internal validation of the 8-feature logistic regression model (B = 1000, *n* = 40). (A) Apparent vs. optimism-corrected AUROC; (B) Bootstrap optimism distribution; (C) Distribution of optimism-corrected AUROC.
