# Supplementary material for: Eravacycline in Critically Ill Patients with Multidrug-Resistant Gram-Negative Infections: A Real-World Cohort Study
Source: J Clin Med. 2026 Jul 17;15(14):5631. doi: 10.3390/jcm15145631 (PMC13412459; doi:10.3390/jcm15145631)

Supplementary Materials

1. Bootstrap optimism-corrected internal validation

Cohort size : n = 40
Deceased : 30
Survivors : 10
Features : 27
Bootstrap samples : 1000
Random seed : 42

Table S1 Bootstrap optimism-corrected internal validation summary for all three machine learning models (Logistic Regression, Random Forest, Gradient Boosting; B = 1000, *n* = 40), including apparent AUROC, mean optimism, and corrected AUROC

Model	Apparent AUC	Mean optimism	Optimism	95% CI Optimism-corrected AUC	Corrected 95% CI	.632 AUC	.632+ AUC	Bootstrap iterations
Logistic Regression	1.0	0.078	[0.013, 0.180]	0.922	[0.820, 0.987]	0.835	0.835	1000
Random Forest	1.0	0.039	[0.000, 0.122]	0.961	[0.878, 1.000]	0.854	0.854	1000
Gradient Boosting	1.0	0.099	[0.003, 0.277]	0.901	[0.723, 0.997]	0.795	0.795	1000

Interpretation

Logistic Regression: apparent 1.000 -> corrected 0.922 (optimism +0.078, relative shrinkage +7.8%)

Random Forest: apparent 1.000 -> corrected 0.961 (optimism +0.039, relative shrinkage +3.9%)

Gradient Boosting: apparent 1.000 -> corrected 0.901 (optimism +0.099, relative shrinkage +9.9%)

2. Bootstrap optimism-corrected internal validation for 8-feature logistic regression

Cohort size : n = 40
Events (deceased) : 30
Non-events (survivors) : 10
Features (k) : 8
Events per variable (EPV): 3.75
Bootstrap iterations : 1000
Random seed : 42

Apparent AUROC : 0.873
Mean optimism (AUROC) : +0.097
Optimism-corrected AUROC : 0.776 (95% CI 0.643 - 0.900)
Relative shrinkage : +11.1%
.632 AUROC : 0.771
.632+ AUROC : 0.738

Apparent Brier : 0.107
Corrected Brier : 0.157 (95% CI 0.099 - 0.224)
Apparent cal. slope : 1.314
Corrected cal. slope : 0.121 (95% CI -4.739 - 1.365)
Apparent cal. intercept : -0.230
Corrected cal. intercept : 0.606 (95% CI -0.613 - 3.219)

Table S2 Performance table for the 8-feature logistic regression model, including bootstrap-corrected Brier score, calibration slope, and calibration intercept

n	B (bootstrap)	Metric	Apparent	Optimism (mean)	Optimism-corrected	Corrected 95% CI	0.632	.632+
40	1000	AUROC	0.873	0.097	0.776	[0.643, 0.900]	0.771	0.738
40	1000	Brier score	0.107	-0.05	0.157	[0.099, 0.224]	-	-
40	1000	Calibration slope	1.314	1.193	0.121	[-4.739, 1.365]	-	-
40	1000	Calibration intercept	-0.23	-0.836	0.606	[-0.613, 3.219]	-	-

Table S3 Odds ratio table for the 8-feature logistic regression model

Variable	Type	Beta	Odds ratio	OR 95% CI
Age (years)	continuous (per 1 SD)	0.25	1.283	[0.681, 3.478]
APACHE II	continuous (per 1 SD)	0.994	2.702	[1.019, 6.293]
SOFA	continuous (per 1 SD)	0.515	1.674	[0.813, 3.278]
ARDS	binary	0.185	1.203	[0.601, 2.560]
Vasopressor use	binary	0.554	1.74	[0.689, 3.688]
Eravacycline duration (days)	continuous (per 1 SD)	-0.11	0.896	[0.329, 2.075]
ICU day eravacycline started	continuous (per 1 SD)	0.505	1.656	[0.878, 3.543]
Polymicrobial infection	binary	0.378	1.46	[0.580, 3.351]

Supplementary Table S4. Full antimicrobial susceptibility profiles for 25 isolates with available data.

	Organism	Specimen	Eravacycline	Cefiderocol	Meropenem	Imipenem	Colistin	Tigecycline	Aminoglycosides	Fluoroquinolones
1	*K. pneumoniae*	Urine	S	S	**R**	**R**	**R**	ND	**R**	**R**
2	*K. pneumoniae*	Peritoneal fluid	S	S	**R**	**R**	**R**	ND	**R**	**R**
3	*K. pneumoniae*	Wound secretion	S	S	**R**	**R**	**R**	ND	**R**	**R**
4	*K. pneumoniae*	Catheter tip / Wound	S	**R**	**R**	**R**	**R**	ND	**R**	**R**
4	*A. baumannii*	Catheter tip / Wound	S	S	**R**	**R**	S	ND	**R**	**R**
4	*P. aeruginosa*	Wound secretion	S	S	**R**	**R**	S	ND	**R**	**R**
5	*K. pneumoniae*	Bronchial aspirate	S	S	**R**	**R**	I	ND	**R**	**R**
6	*A. baumannii*	Bronchial aspirate	S	ND	**R**	**R**	S	ND	**R**	**R**
7	*K. pneumoniae*	Peritoneal fluid / Urine	S	ND	**R**	**R**	**R**	**R**	**R**	**R**
8	*K. pneumoniae*	Bronchial aspirate	S	S	**R**	**R**	**R**	ND	**R**	**R**
8	*A. baumannii*	Bronchial aspirate	S	**R**	**R**	**R**	**R**	ND	**R**	**R**
9	*K. pneumoniae*	Bronchial aspirate	S	S	**R**	**R**	**R**	**R**	**R**	**R**
10	*K. pneumoniae*	Bronchial aspirate	S	**R**	**R**	**R**	**R**	**R**	**R**	**R**
10	*A. baumannii*	Bronchial aspirate	S	S	**R**	**R**	I	ND	**R**	**R**
11	*A. baumannii*	Bronchial aspirate / Urine	S	ND	**R**	**R**	I	ND	**R**	**R**
12	*K. pneumoniae*	Wound secretion	S	ND	**R**	**R**	S	ND	**R**	I
12	*P. aeruginosa*	Wound secretion	S	ND	S	I	S	ND	S	I
13	*K. pneumoniae*	Bronchial aspirate	S	S	**R**	**R**	**R**	ND	**R**	**R**
14	*K. pneumoniae*	Bronchial aspirate	S	ND	**R**	**R**	S	**R**	**R**	**R**
15	*K. pneumoniae*	Bronchial aspirate	S	ND	**R**	**R**	**R**	S	S	**R**

	Organism	Specimen	Eravacycline	Cefiderocol	Meropenem	Imipenem	Colistin	Tigecycline	Aminoglycosides	Fluoroquinolones
15	*P. aeruginosa*	Bronchial aspirate	S	ND	**R**	**R**	S	ND	**R**	**R**
16	*K. pneumoniae*	Bronchial aspirate	S	S	**R**	**R**	**R**	**R**	**R**	**R**
16	*Stenotrophomonas maltophilia*	Bronchial aspirate	S	S	**R**	**R**	ND	ND	ND	ND
17	*K. pneumoniae*	Wound secretion	S	ND	**R**	**R**	**R**	**R**	S	**R**
18	*K. pneumoniae*	Blood culture	S	S	**R**	**R**	**R**	**R**	**R**	**R**
19	*K. pneumoniae*	Urine	S	**R**	**R**	**R**	**R**	**R**	**R**	**R**
20	*P. aeruginosa*	Wound secretion	S	S	**R**	**R**	**R**	ND	**R**	**R**
20	*K. pneumoniae*	Wound secretion	S	**R**	**R**	**R**	**R**	**R**	**R**	**R**
20	*A. baumannii*	Wound secretion	S	S	**R**	**R**	S	ND	**R**	**R**
21	*A. baumannii*	Bronchial aspirate	S	S	S	S	S	ND	S	I
21	*Enterobacter cloacae*	Bronchial aspirate	S	ND	S	S	ND	S	S	S
22	*P. aeruginosa*	Bronchial aspirate	S	S	**R**	**R**	**R**	ND	**R**	**R**
23	*A. baumannii*	Bronchial aspirate	S	**R**	**R**	**R**	S	ND	**R**	**R**
23	*K. pneumoniae*	Bronchial aspirate	S	**R**	**R**	**R**	**R**	ND	**R**	**R**
24	*K. pneumoniae*	Bronchial aspirate	S	S	**R**	**R**	**R**	ND	**R**	**R**
24	*Providencia stuartii*	Bronchial aspirate	S	S	**R**	**R**	**R**	ND	**R**	**R**

	Organism	Specimen	Eravacycline	Cefiderocol	Meropenem	Imipenem	Colistin	Tigecycline	Aminoglycosides	Fluoroquinolones
25	*K. pneumoniae*	Wound secretion	S	ND	**R**	**R**	**R**	ND	**R**	**R**

Abbreviations: S, susceptible; R, resistant; I, intermediate (susceptible at increased exposure, per EUCAST); ND, not determined/not tested. Eravacycline susceptibility was confirmed by dedicated disk diffusion testing (PS-14) in all cases. Aminoglycoside results represent amikacin and/or gentamicin. Fluoroquinolone results represent ciprofloxacin and/or levofloxacin. All susceptibility interpretations follow EUCAST breakpoints.

Figure S1. Internal validation of the 8-feature logistic regression model (B=1000, n=40) (A) Apparent vs. optimism-corrected AUROC (B) Bootstrap optimism distribution (C) Distribution of optimism- corrected AUROC.

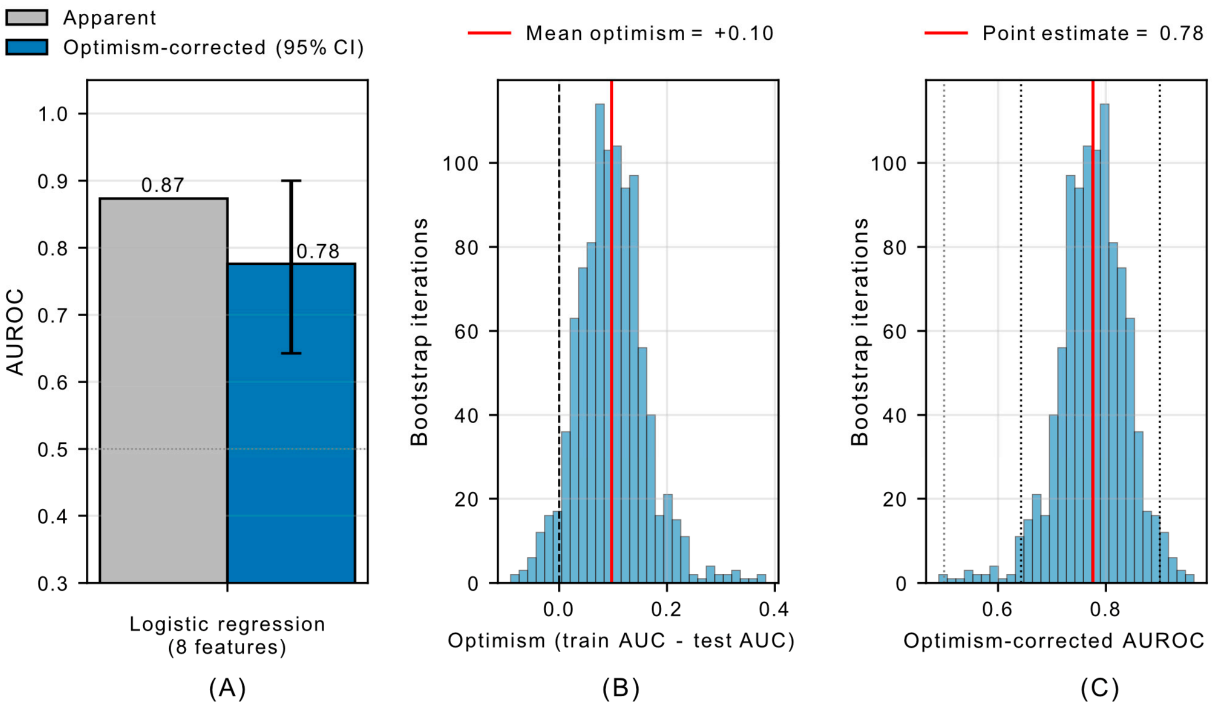

Supplement: Supplementary file 1 [file jcm-15-05631-s001.zip › jcm-4377639-supplementary.pdf]
